# Dynamic Python-Based Method Provides Quantitative Analysis of Intercellular Junction Organization During *S. pneumoniae* Infection of the Respiratory Epithelium

**DOI:** 10.3389/fcimb.2022.865528

**Published:** 2022-06-10

**Authors:** Devons Mo, Shuying Xu, Juan P. Rosa, Shakir Hasan, Walter Adams

**Affiliations:** ^1^ Department of Biological Sciences, San Jose State University, San Jose, CA, United States; ^2^ Department of Molecular Biology and Microbiology, Tufts University, Boston, MA, United States; ^3^ Graduate Program in Immunology, Tufts Graduate School of Biomedical Sciences, Boston, MA, United States; ^4^ Department of Biology, University of Puerto Rico, Cayey, PR, United States; ^5^ Institute of Microbiology of the CAS, Prague, Czechia

**Keywords:** adherens junctions, tight junctions, image analysis, brightness normalization, pneumonia

## Abstract

Many respiratory pathogens compromise epithelial barrier function during lung infection by disrupting intercellular junctions, such as adherens junctions and tight junctions, that maintain intercellular integrity. This includes *Streptococcus pneumoniae*, a leading cause of pneumonia, which can successfully breach the epithelial barrier and cause severe infections such as septicemia and meningitis. Fluorescence microscopy analysis on intercellular junction protein manipulation by respiratory pathogens has yielded major advances in our understanding of their pathogenesis. Unfortunately, a lack of automated image analysis tools that can tolerate variability in sample-sample staining has limited the accuracy in evaluating intercellular junction organization quantitatively. We have created an open source, automated Python computer script called “Intercellular Junction Organization Quantification” or IJOQ that can handle a high degree of sample-sample staining variability and robustly measure intercellular junction integrity. *In silico* validation of IJOQ was successful in analyzing computer generated images containing varying degrees of simulated intercellular junction disruption. Accurate IJOQ analysis was further confirmed using images generated from *in vitro* and *in vivo* bacterial infection models. When compared in parallel to a previously published, semi-automated script used to measure intercellular junction organization, IJOQ demonstrated superior analysis for all *in vitro* and *in vivo* experiments described herein. These data indicate that IJOQ is an unbiased, easy-to-use tool for fluorescence microscopy analysis and will serve as a valuable, automated resource to rapidly quantify intercellular junction disruption under diverse experimental conditions.

## 1 Introduction

A growing area of research has documented how pathogens damage the respiratory epithelium, an important physical barrier that prevents infectious microbes that enter the airways from disseminating into the bloodstream and gaining access to the deeper host tissues. Integral to the success of this barrier function are different intercellular junctions (IJs) such as adherens junctions (AJs) and tight junctions (TJs) (Campbell et al., 2017). An important feature shared by AJs and TJs is their distinct localization pattern between the borders of epithelial cells (Garcia et al., 2018). Both AJs and TJs form extracellular connections between adjacent cells and play important roles to maintain the structural and functional integrity of the respiratory epithelium (Ganesan et al., 2013). AJs mediate cell-cell adhesion and consist of several proteins, including E-cadherin, nectin, catenins, and afadin (Takeichi, 2014). Meanwhile, tight junctions regulate the passage of small molecules between cells, establish cell polarity, and are comprised of occludins, claudins, and junctional adhesion molecules (Zihni et al., 2016). Therefore, accurate and repeatable quantification of the integrity of AJs and TJs under various experimental conditions is an important aspect to many fields of study.

One area where the characterization of IJ integrity has had a large impact has been in understanding virulence strategies of respiratory bacterial pathogens that disrupt IJs as an important aspect of the disease process using fluorescence microscopy analysis. This is well documented by investigations of the Gram-positive pathogen, *Streptococcus pneumoniae*, which remains the leading cause of pneumonia mortality worldwide (Troeger et al., 2018). Investigation of IJ disruption during *S. pneumoniae* infection has revealed evidence of TJ disruption (Rayner et al., 1995), including fluorescence microscopy analysis of pneumococcal infection of human cells, which revealed reduced levels of the TJ proteins occludin, ZO-1, and claudin-5 (Peter et al., 2017; Jacques et al., 2020). Similar results were found after infection with the Gram-positive pathogen *Bordetella pertussis*, which led to decreased levels of ZO-1, occludin, and TJ organization, all of which were exacerbated by the production of adenylate cyclase toxin-hemolysin (Hasan et al., 2018). These studies demonstrate how qualitative and quantitative fluorescence microscopy analysis can provide important insights on how respiratory bacteria invade the host epithelium.

While qualitative assessments of IJ disruptions to the respiratory epithelium offer some insight into the pathogenesis of a disease, quantitative measurements of the arrangement of IJs can provide more comprehensive assessments of perturbations during infections. An image analysis metric commonly used to assess IJ disruption is mean fluorescence (McNeil et al., 2006; Hasan et al., 2018). While this metric can serve as a proxy measurement for IJ health, this analysis assumes that the expression of IJ proteins always has a strong linear correlation with the arrangement of IJs. However, depending on the context of the infection, IJ disruption can be observed as a rearrangement of IJ proteins rather than the destruction of these proteins. For instance, *Helicobacter pylori* infection of host cells recruits ZO-1 to extrajunctional sites of bacterial attachment (Amieva et al., 2003). Likewise, *Neisseria meningitidis* infection of brain endothelial cells recruited AJ and TJ proteins to bacterial attachment sites and induced what were termed “ectopic early junction-like domains” (Mathieu et al., 2009). In these cases, even though IJ disruption was qualitatively observed, this disruption is not captured by mean fluorescence, alone, thus limiting the application of this quantitative metric.

To circumvent the reliance on using mean fluorescence in image analysis, other methods have been developed which quantify the arrangement of IJs. Several methodologies utilize a semi-automated approach where the measurement and analysis protocols are augmented with computer scripts (Terryn et al., 2013; Brezovjakova et al., 2019; Gray et al., 2020). These methodologies usually require moderate human input for parameter set-up or data formatting, with the remainder of the analysis being completed by automation. Successful application of these methodologies requires proficiency in 1) using the automated scripts, 2) interpreting script readouts, and 3) adjusting script-specific parameters when quantitative and qualitative sample readouts do not match. Thus, semi-automated analysis methodologies are still relatively low-throughput and subject to human error and bias, especially if the user is inadequately trained.

Fully automated image analysis algorithms improve upon semi-automated methodologies as they dramatically increase the speed of analysis and minimize the opportunity for human bias. However, one major challenge that fully automated algorithms must overcome when analyzing microscopy images is how to successfully control for the variation in brightness for a signal of interest. These variations in brightness can occur when there are minor deviations in the optimal z-plane to detect a signal (i.e. a sample is not perfectly flat) and is particularly relevant when attempting to quantify IJs that are not present along the entire vertical border of lung epithelial cells. For example, TJs only occupy the most apical position along the vertical border between lung epithelial cells. Likewise, AJs are located immediately underneath the TJs in the basolateral direction of this vertical border. Because these IJs localize to specific vertical planes in a sample, the presence of even minor z-plane deviations can result in substantial variations in signal brightness. Therefore, finding a way to normalize this signal variability prior to quantifying IJ disruption is crucial for a fully automated image analysis algorithm to function reliably.

Here we describe a novel, fully automated, image analysis Python script for the quantification of IJ integrity. Notably, this script performs a normalization step prior to quantification, which facilitates the reliable analysis of IJ disruption. Due to this normalization step, the predetermined parameters for quantification are more consistently optimal, and artifacts are minimized. The Python script was designed to quantify the organization of AJs and TJs that form on polarized epithelial monolayers and as such we term this script “Intercellular Junction Organization Quantification” or IJOQ. In this study we will discuss the development process of this script and demonstrate its applications to *in vitro* and *in vivo* models of *S. pneumoniae* infection and *B. pertussis* infection of human bronchial epithelial cells.

## 2 Materials and Methods

### 2.1 Preparation of Epithelial Monolayers

Human pulmonary mucoepidermoid carcinoma-derived NCI-H292 (H292) cells were prepared as previously described (Adams et al., 2020). Briefly, H292s were grown on the underside of collagen-coated Transwell filters (0.33-cm^2^, Corning Life Sciences) in RPMI 1640 medium (ATCC) with 2 mM L-glutamine, and 10% FBS.

### 2.2 Bacterial Strains and Growth Conditions

*S. pneumoniae* strain TIGR4 (serotype 4) was grown in Todd-Hewitt broth (BD Biosciences) supplemented with 0.5% yeast extract in 5% CO_2_ and Oxyrase (Oxyrase, Mansfield, OH) and used at late log phase. For mice experiments, bacteria were grown to log phase and diluted in PBS to appropriate concentrations, as required. Bacterial number in stocks was confirmed by plating serial dilutions on blood agar.

### 2.3 Handling of Animals

Wild type BALB/cJ mice were purchased from The Jackson Laboratory (Bar Harbor, ME). Mice were matched for age and sex and maintained in a specific pathogen-free facility at Tufts University. All procedures were performed in accordance with Institutional Animal Care and Use Committee approved protocols.

### 2.4 Disruption of H292 Monolayer With Adherens Junction Inhibitor

H292 monolayers were treated with 10 mM 1,4-dithiothreitol (DTT) (Millipore Sigma) and incubated for 3 hours at 37°C. After incubation, monolayers were washed in PBS+Ca/Mg. To prepare samples for confocal imaging, monolayers were fixed in 4% paraformaldehyde and stored at 4°C. Monolayers were permeabilized with 0.1% Triton X-100 in PBS plus 3% BSA. Then, the monolayers were stained with DAPI, phalloidin, and primary anti-E-cadherin antibody (24E10, Cell Signaling), followed by a secondary α-rabbit-FITC antibody (Molecular Probes). The monolayers were then excised from the transwell inserts and mounted onto glass slides with ProLong Gold (ThermoFisher). Samples were visualized with confocal microscopy (Zeiss LSM 700).

### 2.5 H292 Monolayer Treatment and Sample Preparation

The apical surfaces of H292 monolayers were infected with 1 x 10^6^ or 1 x 10^7^ bacteria and incubated for 2.5 hours at 37°C. After incubation, monolayers were washed in PBS+Ca/Mg. Monolayers were fixed in 4% paraformaldehyde and stored at 4°C, then were permeabilized with 0.1% Triton X-100 in PBS plus 3% BSA. The monolayers were stained with DAPI, phalloidin, and primary anti-E-cadherin antibody (24E10, Cell Signaling), followed by a secondary α-rabbit-FITC antibody (Molecular Probes). The monolayers were then excised from the transwell inserts and mounted onto glass slides with ProLong Gold (ThermoFisher). Samples were visualized with confocal microscopy (Leica SP8).

### 2.6 Mouse Treatment and Sample Preparation

Mice were challenged intratracheally with 1 x 10^7^ bacteria or mock-infected with PBS. At 3- and 6-hours post-infection, mice were sacrificed, and their lungs were harvested. Lung tissues were fixed in 4% paraformaldehyde and sectioned to a thickness of 250 μm with a Leica vibratome (0.145 mm/s, 70 Hz, blade angle 5°). Lung sections were permeabilized with 0.1% Triton X-100 in PBS plus 3% BSA, then stained with the primary anti-E-cadherin antibody (24E10, Cell Signaling) overnight. Following this, the lung sections were stained with DAPI, phalloidin, and secondary α-rabbit-FITC antibody. Samples were mounted with Vectashield Antifade Mounting Medium (Vector Laboratories) and visualized with confocal microscopy (Leica SP8).

### 2.7 Computer Simulation of Intercellular Junction Disruption

To assist in the *in silico* validation of the IJOQ Python script, a separate Python script, termed the “Simulator” script, was written to create simulations of IJ disruption. The script is open access, downloadable and has been deposited in the IJOQ project repository on GitHub (GNU General Public License version 3.0; https://github.com/DevonsMo/IJOQ/blob/main/Simulator.py) When executed, the simulator script produces simulated images of cell monolayers under various degrees of IJ disruption. To simulate IJ disruption, a probability is applied to each line to remove the line from the simulated image. Probabilities of 0, 0.1, 0.2, 0.3, 0.5, and 0.7 were used to simulate varying degrees of IJ disruption. Because a random number of lines are removed for each simulated image, the degree of removal is recorded as a sum of the lengths of the remaining lines, or the total junction length. The image is then saved as a completed simulated image.

### 2.8 Development of a Python Script to Measure Adherens Junction Health

**Figure 1:**
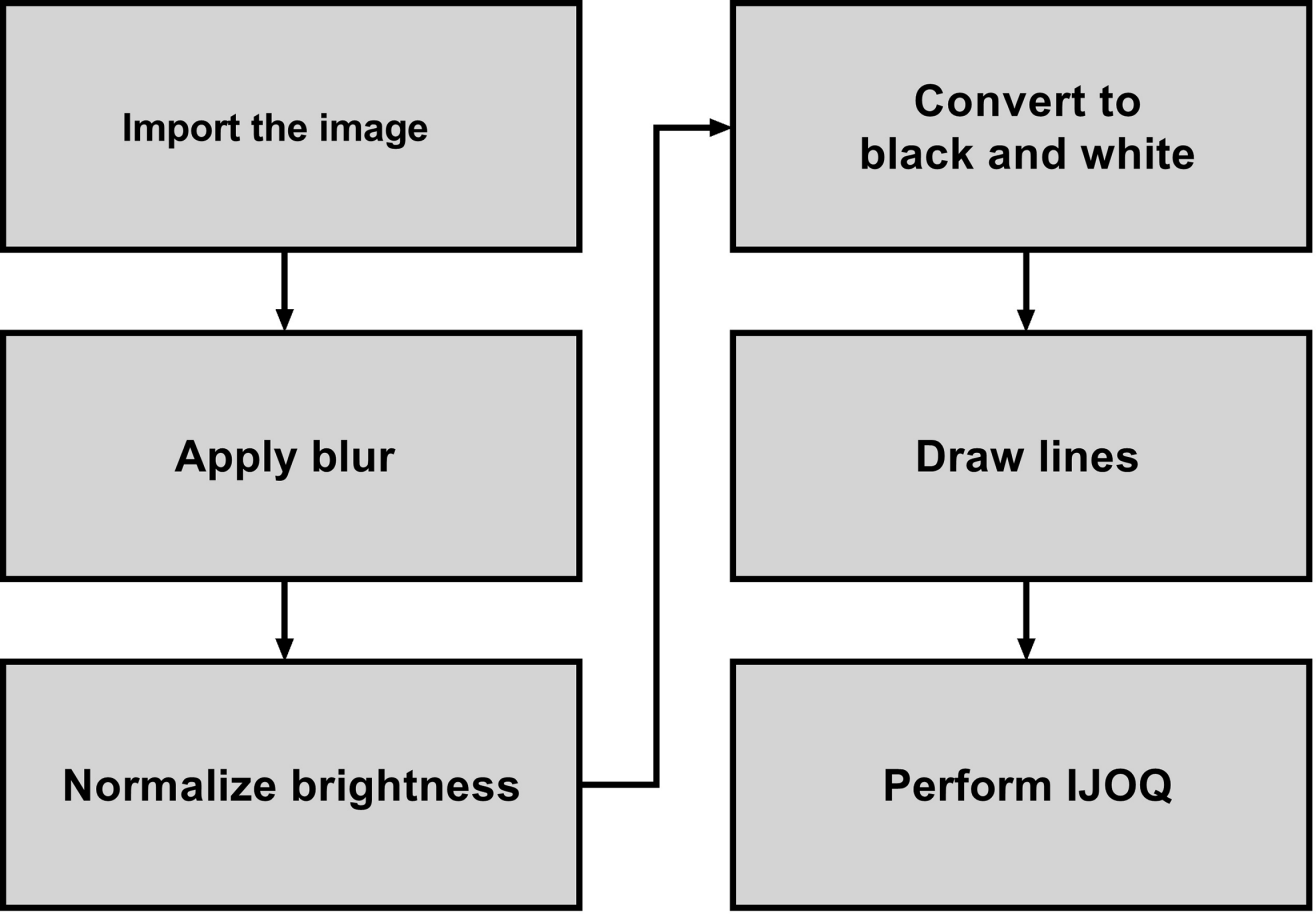
Flowchart which depicts the steps used to analyze the image for AJ health.

A novel Python script called “Intercellular Junction Organization Quantification” or IJOQ was developed in order to automate the measurement of IJ disruption in epithelial cells. This script was designed to accept an image or a batch of images, process each image, measure the IJ organization, and then save the data in a.csv file. A flowchart summarizes the steps used by the script to analyze each image (**Figure 1**).

#### 2.8.1 Importing the Image

After each image is imported, the image is resized such that the average of the height and width of the image is 512 pixels. This compression step reduces processing time in later steps, as well as ensures that the resulting IJOQ value is standardized. The aspect ratio of the image is conserved during compression.

Because there is a potential for the imported image to be a composite that contains multiple pseudo-colored fluorescent channels, the green color channel is extracted and used for further image processing. The script may be instructed to extract blue, red, or white color channels instead during calibration.

A mild Gaussian blur with a radius of 5 pixels is applied to the extracted channel in order to remove the effects of noise on later steps. The script may be instructed to use a different blur radius during calibration, depending on the extent of the noise present.

#### 2.8.2 Normalizing the Brightness

To control for the substantial variations in brightness that exist in confocal microscopy images due to minor deviations in the z-plane, a normalization step was created to equalize the brightness within and across confocal microscopy images.

##### 2.8.2.1 Measuring Background Brightness

**Figure 2:**
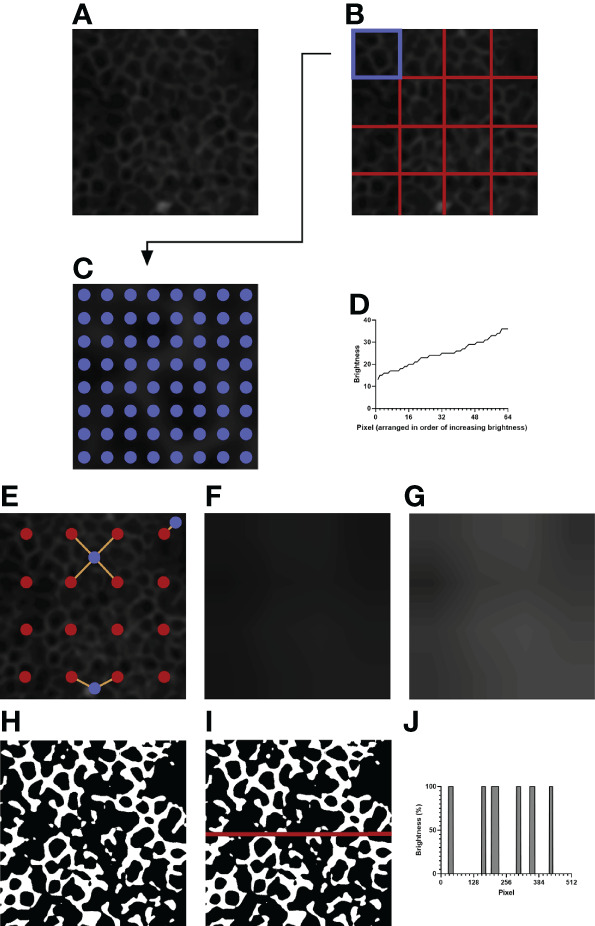
An example of image processing. **(A)** The initial image after resizing and blurring. The green channel has been extracted. **(B)** The image is divided into 4 x 4 equally sized sections. **(C)** Depicted is the top left section of **(B)**, highlighted in blue. 8 x 8 evenly spaced pixels, highlighted in blue, are sampled for their brightness values. **(D)** The brightness values of the sampled pixels are arranged in increasing order. The brightness value of the 35^th^ pixel is taken as the normalization threshold value of the section. **(E)** Example pixels are highlighted in blue. Orange lines are drawn between each example pixel and their nearby section centers, marked in red. **(F)** The expected brightness map is created by calculating the distance-based weighted average of the normalization threshold values of nearby sections for each pixel in the image. The map predicts the background brightness value of a given pixel. **(G)** The brightness map in **(F)** is enhanced in Adobe Photoshop CC 2021 to improve visibility. The enhanced version is not used for analysis and is shown to highlight the differences in brightness in the image. **(H)** Pixels that are brighter than their expected brightness values are set to white, while all other pixels are set to black. **(I)** IJOQ analysis generally traces 10 horizontal and 10 vertical lines through the prepared image. An example line is shown on the image. **(J)** The brightness values of the pixels traced through the example line in **(I)** are measured. Each color change (i.e. vertical line) increments the cell border frequency by 0.5.

To correct for within-image brightness variations, the image is first divided into equally sized sections. A larger section size, due to sampling a wider area, decreases the ability to normalize the within-image variation; however, a smaller section size, due to its limited coverage, decreases the ability to discriminate between the background and the IJs. Furthermore, smaller sections increase the computational time, as more pixels must be sampled overall. Dividing the image (**Figure 2A**) into 4 x 4 sections (**Figure 2B**) was qualitatively determined to be most optimal.

For a given section (i.e. **Figure 2B**, the upper leftmost square, highlighted in blue) the brightness values of 8 x 8 equally spaced pixels are recorded (**Figure 2C**), then arranged in order of increasing brightness (**Figure 2D**). The 35^th^ pixel was determined to be an adequate threshold of background brightness for H292 cell monolayers during calibration (See **2.10 Calibration of the IJOQ Script**), and the brightness value of this pixel is recorded as the normalization threshold value of its section. Because there are 16 sections this results in a total of 16 normalization threshold values.

##### 2.8.2.2 Removing Background Brightness

The 16 normalization threshold values can be used to estimate the brightness value of the background in each section. However, because normalization threshold values are not necessarily similar, when normalization threshold values are applied uniformly to all pixels in their sections, there will be noticeable discrepancy in brightness along the borders of adjacent sections, leading to artifacting. To remove the potential for artifacting, the expected background brightness value of each pixel is calculated individually by taking into consideration the normalization threshold values of all nearby sections.

First, because the sampled pixels are distributed uniformly across a section, it is assumed that the normalization threshold value of a given section best applies to the center pixel of that section. Therefore, the center pixel of each section is given the expected background brightness value equivalent to the normalization threshold value of its section.

Then, the remaining pixels are considered. For all pixels which do not lie at the center of their section, there can be up to four section centers which are both within a section’s height vertically and a section’s width horizontally from that pixel: to the upper left, upper right, lower left, and lower right (**Figure 2E**, See blue pixel connected to four red pixels). The estimated background brightness value of a given pixel is calculated by linearly scaling the normalization threshold values of the four sections, based on the pixel’s horizontal and vertical distance to the centers of those four sections. The equations used to calculate the expected brightness value of a given pixel are shown below:


$$Displacement_{x}=Position_{pixel,x}-Position_{\sec tion\;center,\;top\;left,x}$$

$$Displacement_{y}=Position_{pixel,y}-Position_{\sec tion\;center,\;top\;left,y}$$

$$Threshold_{average,top}=\left(\frac{Displacement_{x}}{Width}\times Threshold_{top\;left}\right)+\left(\left(1-\frac{Displacement_{x}}{Width}\right)\times Threshold_{top\;right}\right)$$

$$Threshold_{{}_{average,bottom}}=\left(\frac{Displacement_{x}}{Width}\times Threshold_{bottom\;\;left}\right)+\left(\left(1-\frac{Displacement_{x}}{Width}\right)\times Threshold_{bottom\;right}\right)$$

$$Brightness_{\exp ected}=\left(\frac{Displacement_{y}}{Height}\times Threshold_{average,\;top}\right)+\left(\left(1-\frac{Displacement}{Height}\right)\times Threshold_{average,bottom}\right)$$

This final calculated value, which effectively takes a horizontal displacement- and vertical displacement-based weighted average of the normalization threshold values of the nearest four sections, provides the expected brightness value of a pixel if that pixel does not contain an IJ.

Pixels which are near the border of the image will have fewer than four section centers that are near enough to be considered for calculation (**Figure 2E**, see blue pixels connected to one or two red pixels). Therefore, if fewer than four section sections are detected for a given pixel, the normalization threshold values of the missing sections are substituted by those of the sections nearest to the missing section centers.

**Figure 2F** depicts the brightness map, an image which shows the expected background brightness values when the process is repeated for every pixel in the image. Because the differences present in the brightness map are subtle to the human eye, **Figure 2G** depicts an equivalent brightness map enhanced with Adobe Photoshop CC 2021 using the Brightness/Contrast adjustment feature with a brightness setting of +150 and a contrast setting of +50. While this enhanced brightness map is not part of the normalization process, it highlights to the observer the areas in which brightness variations exist. To remove the background, the expected background brightness value of each pixel is subtracted from the brightness value of that pixel. To reduce noise, an extra value, calculated by taking 10% of the expected background brightness value, is further subtracted from the brightness value of the pixel. This value can be adjusted during calibration, depending on the extent of the noise present. An additional filtering step is then performed to increase the contrast of the resulting image. Pixels whose brightness values remained positive after normalization are set to white, while pixels whose brightness values became 0 or negative are set to black, resulting in the final prepared image that is now ready for analysis (**Figure 2H**).

#### 2.8.3 Performing IJOQ

IJs are expected to lie at cell borders, so they should resemble a mesh-like structure when intact and healthy. Therefore, quantifying the organization of IJs should revolve around confirming this mesh-like appearance. To this end, the IJOQ is calculated by considering the number of color changes – from black to white and from white to black – that are detected when a path is traced through the image. For example, a single line traced horizontally through an image that has been prepared for analysis will encounter several color changes across the pixels present in the path (**Figure 2I**). Correspondingly, these color changes across pixels are represented as changes in percent brightness (**Figure 2J**). Any time there is a change in percent brightness is detected, the IJOQ script assumes that the path is either entering a border or exiting a cell border. Therefore, the IJOQ script increments the “cell border frequency” value of the line by 0.5. As a result, should a line cross an entire intact cell border, which is detected as a color change from black to white to black, the value of the cell border frequency of the line will increase by 1. An organized network of IJs is expected to have a greater cell border frequency than a disorganized or disrupted network because any path traced through an image is expected to detect more cell borders in an intact network.

10 evenly spaced horizontal lines are traced across the width of the image and 10 evenly spaced vertical lines are traced across the height of the image. The cell border frequencies of all 20 paths are summed and divided by the total length of all lines. The resulting number, the IJOQ, expresses the number of expected intact cell borders that are crossed per pixel traced through the image. If a single image was imported, this value is then printed to the user; otherwise, if a batch of images was imported, the IJOQ values of all images are saved into a.csv file at the conclusion of analysis.

### 2.9 Installation and Setup of the IJOQ Script

The IJOQ script and all relevant scripts are open access, downloadable, and have been deposited in Github (GNU General Public License version 3.0; https://github.com/DevonsMo/IJOQ/releases). First, the latest version of Python 3 should be installed and the latest version of IJOQ should be downloaded. Following this, Python libraries required for the IJOQ computer script are installed by executing the “IJOQ Setup.py” script. Upon execution, the IJOQ setup script will update the Python package installer, pip, then install Pillow, NumPy, and Tkinter. The IJOQ script is compatible to run on Windows and Mac.

### 2.10 Calibration of the IJOQ Script

Before analysis, a calibration is performed to ensure that the parameters of the IJOQ script are adequate for the samples. Calibration is performed once, and the parameters derived during calibration can be re-used for later samples, given that the same cell type and imaging settings are used.

Calibration can be performed by executing the “IJOQ Calibration.py” script. The script will prompt for three negative control images, as well as for analysis settings. The calibration script applies a blur to the images, then uses Otsu’s Method to determine a valid threshold for background brightness. The settings can be saved to a.txt file for later usage.

### 2.11 Statistical Analysis

Statistical analysis was performed using GraphPad Prism (GraphPad Software, San Diego, CA). Pearson’s Correlation was calculated for the simulated image analysis. Comparison of the DTT treatment was performed using an unpaired t-test. All other comparisons between two groups were performed using ANOVA with Tukey’s *post-hoc*. P values < 0.05 were considered significant in all cases. Cohen’s d was calculated for the effect size of the DTT treatment, while η^2^ was calculated for the effect size of all other comparisons between groups.

## 3 Results

### 3.1 IJOQ Robustly Analyzes *In Silico* Disruption of IJs

**Figure 3:**
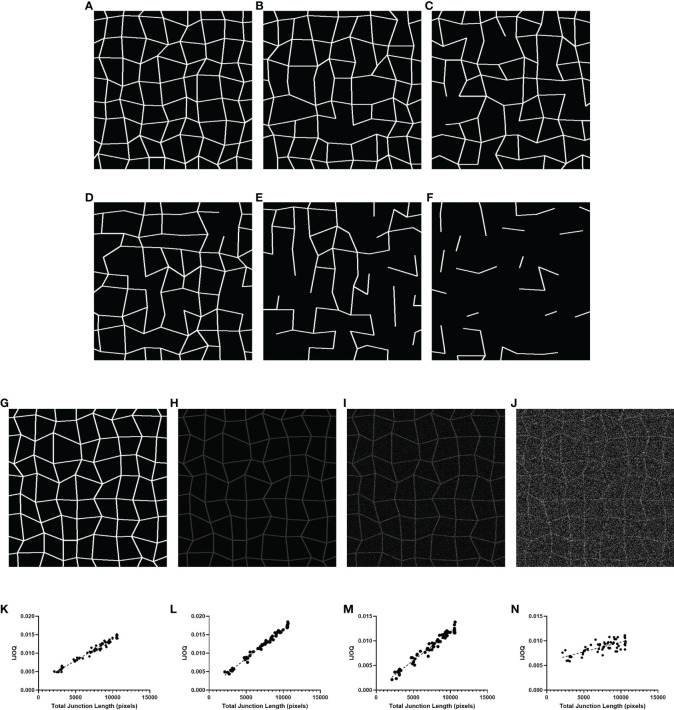
IJOQ versus total junction length in simulated images. Examples of simulated cell monolayer images. IJ disruption was simulated at **(A)** 0%, **(B)** 10%, **(C)** 20%, **(D)** 30%, **(E)** 50%, and **(F)** 70%, respectively. **(G)** An example of the original simulated image with no noise applied. Additive noise was applied to reach **(H)** a signal-to-noise ratio of 10, **(I)** a signal-to-noise ratio of 2 and **(J)** a signal-to-noise ratio of 0.5. IJOQ shows a strong linear correlation under **(K)** ideal conditions (r^2^ = 0.975), **(L)** a signal-to-noise ratio of 10 (r^2^ = 0.989), **(M)** a signal-to-noise ratio of 2 (r^2^ = 0.963) and **(N)** a signal-to-noise ratio of 0.5 (r^2^ = 0.579) as determined by linear regression analysis.

We first sought to determine whether IJOQ analysis is capable of accurately assessing IJ health under optimal, simulated conditions. Therefore, a separate Python script called the “Simulator” (See Materials and Methods) was developed to produce simulated images of IJs in a monolayer under varying degrees of disruption (**Figures 3A–F**). Because this script produces randomized simulations, precise IJ damage is not necessarily equal in all simulated images. As a result, the script records the total length of the IJs as a more precise measure of IJ health.

We produced 60 simulated images, with 10 images each corresponding to an IJ disruption value of approximately 0%, 10%, 20%, 30%, 50% and 70%. The IJOQ script was then used to analyze the simulated images. The IJOQ metric showed significant (r^2^ = 0.975, P < 0.0001) correlation with the total junction length (**Figure 3K**). These findings demonstrate that IJOQ can precisely measure IJ health in a simulated monolayer across a broad range of IJ damage.

In the previous *in silico* experiment, IJOQ analysis was performed on individual simulated monolayers that had varying degrees of IJ disruption, but each monolayer was generated to mimic an ideal circumstance where there is no noise in the image (**Figure 3G**). Because most microscopy images contain various levels of noise we assessed the capacity for the IJOQ script to filter IJ signal from noise by applying IJOQ analysis to images containing suboptimal signal-to-noise ratios. To this end we applied an additive white Gaussian noise to the 60 simulated images and performed IJOQ on the resulting images. The IJOQ metric showed significant correlation (r^2^ = 0.989, p < 0.0001) with the total junction length at a signal-to-noise ratio of 10, which is the approximate signal-to-noise ratio of our images based on preliminary data (**Figures 3H, L**). IJOQ continued to maintain a strong correlation (r^2^ = 0.963, p < 0.0001) at a signal-to-noise ratio of 2 (**Figures 3I, M**), while the strength of the correlation began to decrease at signal-to-noise ratios of less than 0.5. Such signal-to-noise ratios may occur when examining tissues which exhibit high amounts of autofluorescence. For instance, murine renal tissues have previously been reported to display intense autofluorescence at a broad range of excitation wavelengths, including those for DAPI, FITC, and Texas Red (Sun et al., 2011; Zhang et al., 2018). Furthermore, fixatives such as aldehydes are known to increase autofluorescence of fixed tissues and impede histological observation (Baschong et al., 2001). Although IJOQ still exhibited a strong correlation (r^2^ = 0.579, p < 0.0001) at a signal-to-noise ratio of 0.5 (**Figures 3J, N**), we recommend using images with a signal-to-noise ratio of at least 2 because a signal-to-noise ratio of 0.5 could require an impractical number of technical replicates. More crucially, however, because the extent of noise filtering required is determined during calibration, it is important to ensure that the signal-to-noise ratio remains consistent across images for successful application of the IJOQ script.

### 3.2 IJOQ Accurately Detects Chemically Disrupted AJs

**Figure 4:**
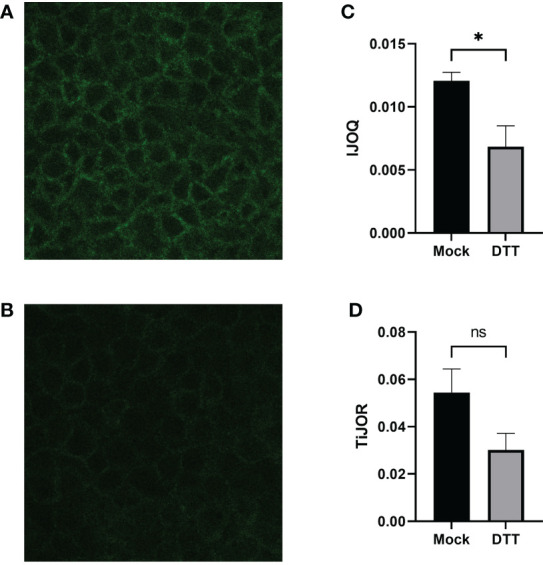
DTT-treated monolayers show significant damage to AJs on a polarized monolayer. Representative images of **(A)** untreated and **(B)** DTT-treated monolayers, respectively. **(C)** IJOQ analysis or **(D)** TiJOR analysis of untreated or DTT-treated monolayers from one experiment performed in quadruplicate. Asterisk indicates intercellular junction organization is significantly greater with mock treatment as determined by an unpaired two-tailed t-test than DTT treatment. Statistical analysis was performed using an unpaired two-tailed t-test. *p < 0.05. ns, not significant.

We next asked whether the IJOQ script would be appropriate for measuring IJ disruption in an established *in vitro* system (Bhowmick et al., 2013; Adams et al., 2020). To address this question, we generated polarized H292 lung epithelial cell monolayers, which express E-cadherin and form AJs (Heijink et al., 2010). The polarized H292 monolayers were then treated for 3 hours with 10 mM DTT, a reagent that has previously been shown to disrupt AJs in cell monolayers by interfering with E-cadherin cell-cell connections (Brückner and Janshoff, 2018). H292 monolayers were stained for E-cadherin and imaged by confocal microscopy to visualize the presence of AJs in the untreated and DTT treated samples (**Figures 4A, B**). Qualitatively we observed substantial disruption to E-cadherin localization and subsequent IJOQ analysis revealed that DTT-treated monolayers exhibited significantly lower AJ organization compared to untreated monolayers (**Figure 4C**, d = 1.15, p < 0.05). These data demonstrate that the IJOQ script successfully detects established chemical methods of AJ disruption.

To assess whether the IJOQ script improved upon semi-automated fluorescence microscopy analysis tools that are already available we reanalyzed our *in vitro* data with one such tool that is commonly used by the research community called Tight Junction Organization Ratio (TiJOR) (Terryn et al., 2013). TiJOR was originally used to quantify TJ organization in response to *Pseudomonas aeruginosa* infection, but has since been used under a diverse range of experimental conditions (Terryn et al., 2013; Putt et al., 2017; Hasan et al., 2018; Schilpp et al., 2021). Briefly, TiJOR operates by having a polygon arbitrarily drawn onto an image of a monolayer. The TiJOR script measures the number of brightness maxima when the drawn polygon is traced. Brightness maxima lower than an arbitrarily chosen threshold value are ignored to remove the effects of noise. Then, the TiJOR value of this polygon can be calculated by dividing the number of brightness maxima by the length of the perimeter of the polygon. The polygon can be expanded by an arbitrary step size, and the new polygon is analyzed similarly. This expansion can be arbitrarily repeated. The final TiJOR value is calculated by averaging the TiJOR values of all analyzed polygons. We performed TiJOR analysis on a set of identical images with a final polygon count of 100 and an expansion step size of 1. In contrast to our IJOQ, TiJOR failed to find a significant difference in AJ organization between the untreated and DTT-treated samples and produced a smaller effect size (**Figure 4D**, IJOQ: d= 1.15 vs. TiJOR: d = 0.91), indicating that IJOQ provides a stronger and more sensitive assessment of chemically induced AJ disruption.

### 3.3 *S. pneumoniae* Infection Causes Dose-Dependent AJ Disruption in Lung Epithelial Cells When Assessed by IJOQ

**Figure 5:**
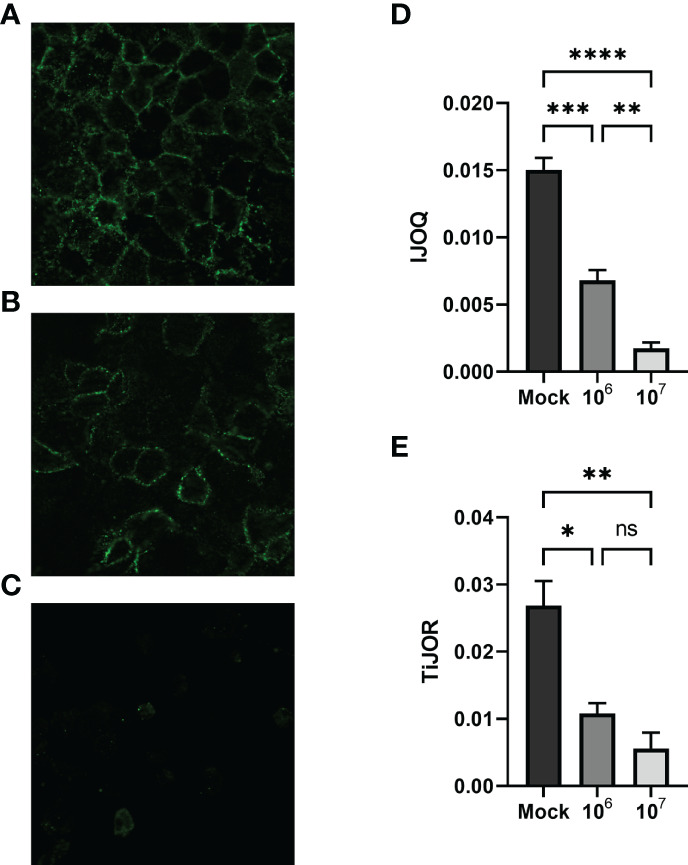
Quantifying AJ health during *S. pneumoniae* infection. Representative images of **(A)** untreated, **(B)** low-dose infection, and **(C)** high-dose infection monolayers, respectively. **(D)** IJOQ analysis or **(E)** TiJOR analysis of untreated or infected monolayers (n = 3). Asterisk(s) indicate intercellular junction organization is significantly greater with mock infection or 1 x 10^6^ as determined by one-way ANOVA with a *post hoc* Tukey test. *p < 0.05, **p < 0.01, ***p < 0.001, ****p < 0.0001. ns, not significant.

Previous research has shown that *S. pneumoniae* infection of lung tissue compromises TJs in lung epithelial cells and AJs in lung endothelial cells; however *S. pneumoniae*-dependent disruption of AJs in lung epithelial cells has yet to be described (Rayner et al., 1995; Peter et al., 2017). To address this question we infected H292 monolayers with *S. pneumoniae* strain TIGR4, a capsular serotype 4 clinical isolate, at low (1 x 10^6^ CFU) and high (1 x 10^7^ CFU) doses. Upon visualizing the monolayers *via* fluorescence microscopy we observed moderate disruption of E-cadherin at the low dose infection and severe disruption of E-cadherin at the high dose infection (**Figures 5A–C**). We quantified these observations by analyzing the images using IJOQ and found that both low and high dose infections exhibited significant AJ disruption (**Figure 5D**, p < 0.001 and P < 0.0001, respectively). Furthermore, IJOQ discriminated between the two infectious doses as the analysis revealed that the high dose disrupted AJs significantly more than the low dose, accurately recapitulating our qualitative observations (**Figure 5D**, p < 0.01). These findings establish that *S. pneumoniae* infection disrupts AJs in lung epithelial cells *in vitro* and underscores the capacity for IJOQ to identify distinct levels of damage between different treatment conditions.

To determine if IJOQ offered greater quantitative resolution of *S. pneumoniae-*mediated AJ disruption than TiJOR analysis, the identical set of images was reanalyzed by TiJOR. Consistent with IJOQ, TiJOR found significant AJ disruption in the low-dose infection (p < 0.05) and high-dose infection (p < 0.01). Importantly, and in contrast to IJOQ, TiJOR failed to discover a significant difference between the low- and high-dose infections (**Figure 5E**, p = 0.40). Additionally, IJOQ analysis offered improved effect sizes when compared with TiJOR analysis (IJOQ: η^2^ = 0.97 vs. TiJOR: η^2^ = 0.85, respectively). These results highlight that compared to TiJOR, IJOQ analysis discriminates between more levels of *S. pneumoniae*-dependent AJ disruption and provides a more robust quantitative assessment of this damage.

### 3.4* S. pneumoniae* Infection Causes Time-Dependent AJ disruption *In Vivo* by IJOQ Analysis

**Figure 6:**
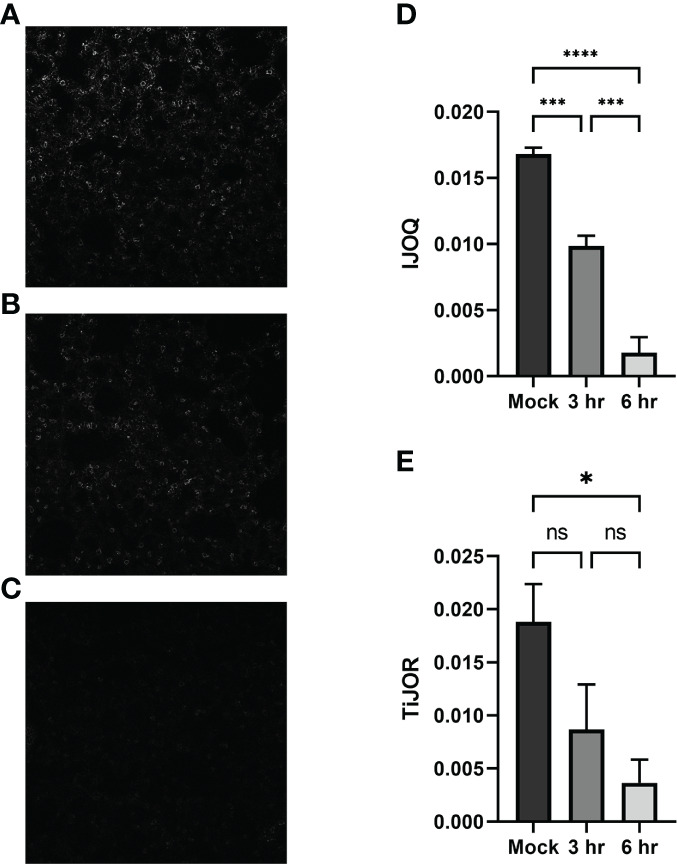
Analysis of *in vivo* effects of *S. pneumoniae* infection on AJs. Representative images of **(A)** untreated, **(B)** 3-hour post-infection, and **(C)** 6-hour post-infection from murine lung alveoli respectively. **(D)** IJOQ analysis or **(E)** TiJOR analysis of lung images (n = 3). Asterisk(s) indicate intercellular junction organization is significantly greater with mock infection or 3hr infection as determined by one-way ANOVA with a *post hoc* Tukey test. *p < 0.05, ***p < 0.001, ****p < 0.0001. ns, not significant.

To determine if our *in vitro* findings of *S. pneumoniae*-dependent AJ disruption extend to an *in vivo* system BALB/cJ mice were challenged intratracheally with 1 x 10^7^ CFU of TIGR4 and the mice were sacrificed 3 hours and 6 hours post-infection. Uninfected and infected lung sections were stained for E-cadherin and then visualized by fluorescence microscopy. Qualitatively, the mock-infected lungs exhibited well maintained AJ structure and were clearly defined (**Figure 6A**). In contrast, partial disruption of AJs was observed at 3 hours post-infection and complete loss of AJ signal was observed at 6 hours post-infection (**Figures 6B, C**). To assess whether our IJOQ script was capable of identifying these qualitative degrees of AJ disruption in an *in vivo* setting, we calibrated the IJOQ script to the new sample parameters (See Materials and Methods) and then performed IJOQ analysis on the lung sections. IJOQ analysis was consistent with our qualitative observations, revealing significant disruption at both the 3 hour (P < 0.001) and 6 hour (p < 0.0001) time points. As with previous experiments, IJOQ analysis identified a significant difference between the two infection conditions with the 6 hour time point exhibiting a significant decrease in AJ organization compared to the 3 hour time point, a direct reflection of the qualitative observations (**Figure 6D**, p < 0.001).

To assess if IJOQ outperformed TiJOR analysis in an *in vivo* context, the lung sections were reanalyzed using the TiJOR program. Strikingly, while TiJOR was able to detect AJ disruption at the 6 hour time point (p < 0.05), it failed to detect AJ disruption at the 3 hour time point (**Figure 6E**, p = 0.179). Furthermore, TiJOR was unable to discriminate between the 3 hour and 6 hour time points (**Figure 6E**, p = 0.670) and compared to IJOQ, TiJOR had a smaller effect size (IJOQ: η^2^ = 0.96 vs. TiJOR: η^2^ = 0.55, respectively). Together, these findings indicate that IJOQ quantifies discrete levels of AJ disruption across *in vivo* samples and provides a stronger assessment of these differences compared to TiJOR.

### 3.5 IJOQ Outperforms TiJOR by Identifying CyaA-Dependent and Independent Disruption of TJs During *B. pertussis* Infection

**Figure 7:**
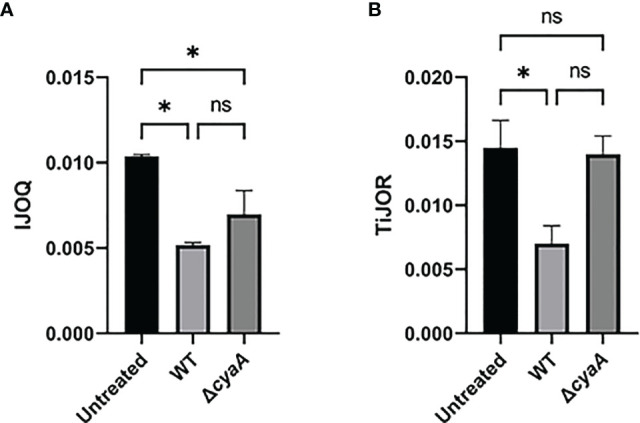
IJOQ outperforms TiJOR analysis of IJ disruption during *B. pertussis* infected bronchial cells. A subset (n = 2) of previously published data from (Hasan et al., 2018) of a *B. pertussis* infection on bronchial epithelial cells was analyzed with **(A)** IJOQ or **(B)** TiJOR. Asterisk indicates intercellular junction organization is significantly greater with mock infection as determined by one-way ANOVA with a *post hoc* Tukey test. *p < 0.05. The original data and analysis can be found in **Figure 1C** from the following publication: Hasan et al. (2018). Bordetella pertussis Adenylate Cyclase Toxin Disrupts Functional Integrity of Bronchial Epithelial Layers. *Infect. Immun.* 86.

While our previous experiments demonstrate that *S. pneumoniae*-dependent AJ disruption can accurately be assessed by IJOQ under *in vitro* and *in vivo* conditions, AJs are only one type of IJ. To explore the breadth of potential IJOQ applications we investigated whether IJOQ would be suitable for the analysis of a different type of IJ (i.e. TJs) in a different experimental system (i.e. *B. pertussis* infection). To this end, we obtained experimental data from a previously published study investigating *B. pertussis* infection of human bronchial epithelial cells cultured using an air-liquid interface model (Hasan et al., 2018). The authors observed that infection with wildtype (WT) *B. pertussis* or an adenylate cyclase toxin mutant (Δ*cyaA*) disrupted ZO-1, a major component of TJs. Notably, after performing TiJOR analysis of entire images the authors demonstrated that while TJ organization was lower in both infectious conditions, only infection with WT bacteria resulted in a significant level of disruption (Hasan et al., 2018, See **Figure 1C**). We calibrated our IJOQ script to the new sample parameters (See Materials and Methods) and analyzed a subset of the original images (**Supplemental Figure 1** and **Table 1**). In agreement with the original study we confirmed that WT *B. pertussis* infection causes significant TJ disruption (**Figure 7A**, η^2^ = 0.93, p < 0.05). Surprisingly, our IJOQ analysis also revealed that the Δ*cyaA* infection causes a significant level of TJ disruption (**Figure 7A**, η^2^ = 0.93, p < 0.05), a finding that is consistent with the original qualitative observations from Hasan et al., but one that they were unable to detect quantitatively with TiJOR. Because we only applied IJOQ to a subset of the original images, we wanted to determine if this subset was still representative of the full data set that was analyzed in the Hasan et al. paper by seeing if we could obtain similar results to the original study using TiJOR. After reanalyzing the same subset of original images with TiJOR we found that WT *B. pertussis* infection decreased TJ organization significantly (p < 0.05, ANOVA with Tukey’s *post-hoc*), while a Δ*cyaA* mutant strain infection did not significantly decrease TJ organization (p = 0.95), recapitulating the original findings of the paper (**Figure 7B**, η^2^ = 0.89, p < 0.05). Taken together, these findings indicate that IJOQ can accurately measure TJ organization in a different experimental system by confirming CyaA-dependent TJ disruption and identifying CyaA-independent TJ disruption during *B. pertussis* infection.

**Table 1:** IJOQ Calibration settings of a *B. pertussis* infection on bronchial epithelial cells.

Parameter Type	Parameter Compressed
image size	512
Channel	1
Blur radius	3
Section size	4
Pixels sampled	8
Normalization cutoff	56
Noise cutoff	0.1
Lines	10

## 4 Discussion

In this study we created an open-source, fully automated, image analysis Python script for the quantification of IJ health. Through a combination of *in silico*, *in vitro*, and *in vivo* assays, IJOQ consistently identified discrete levels of IJ disruption across different experimental systems. This is in part a direct reflection of its core design features which enable it to handle a low signal-to-noise ratio and substantial variations in signal brightness.

The capacity for IJOQ to normalize signal brightness within and across samples through its extensive image pre-processing algorithm is one of the most important properties of the script. Many sources can give rise to variations in signal brightness including minor deviations in the optimal z-plane (e.g. the sample is not perfectly flat), subtle differences in staining protocol, and signal degradation during imaging, among others (Waters, 2009). To accurately account for these variations, IJOQ executes a normalization step to each image prior to quantification (**Figure 2**), which allows for the consistent optimization of thresholding parameters, a substantial reduction in false positive signals, and ultimately a high level of accuracy when assessing IJ disruption. This extensive image pre-processing step distinguishes IJOQ from several of the other semi-automated fluorescence microscopy tools currently available for junction analysis which either have a limited amount of image preprocessing or no preprocessing at all (Terryn et al., 2013; Brezovjakova et al., 2019; Gray et al., 2020). As a result, this could impact the capacity for these scripts to handle high levels of sample-sample variation and detect different levels of IJ disruption. For example, TiJOR mostly refrains from image pre-processing beyond applying a mild Gaussian blur with a radius of 2 pixels (Terryn et al., 2013). Meanwhile, in our study’s comparison between IJOQ and TiJOR across *in vitro* and *in vivo* experiments we found that IJOQ and the corresponding statistical analysis revealed at least one to two discrete levels of IJ disruption in response to infection dose and time that were not detected by TiJOR (**Figures 4** **–** **7**). Furthermore, IJOQ achieved larger effect sizes than TiJOR in every direct comparison of statistical tests performed (**Figures 4** **–** **7**). Incorporation of more extensive pre-processing steps into currently available semi-automated fluorescence microscopy tools may allow users to enhance their dataset analysis and have increased success with more technically challenging assays.

Because IJOQ was originally designed to assist in our analysis of *S. pneumoniae*-dependent disruption of AJs *in vitro*, we were unsure whether it would be able to handle a more challenging *in vivo* experimental system. While successful application of IJOQ to lung sections required the development of a calibration script, IJOQ accurately detected significant E-cadherin disruption *in vivo*, consistent with our *in vitro* results (**Figures 5**, **6**). The finding that *S. pneumoniae* disrupts AJs in lung epithelial cells builds upon a growing body of literature documenting how *S. pneumoniae* infection of lung tissue results in the targeted disruption of IJs (Rayner et al., 1995; Peter et al., 2017; Jacques et al., 2020). Because IJ manipulation is a strategy that is prevalent across a variety of respiratory infections, we assessed whether IJOQ was capable of analyzing IJ disruption caused by a different airborne pathogen in a new experimental system (Hasan et al., 2018). We found that IJOQ analysis of the major TJ protein ZO-1 in bronchial epithelial cells grown using an air liquid interface model was successful in detecting CyaA-dependent and independent pathways of TJ disruption (**Figure 7**). Notably, this finding is consistent with the qualitative observations from the original paper and suggests that multiple *B. pertussis* virulence factors contribute to the disruption of TJs (Hasan et al., 2018). Furthermore, this demonstrates that IJOQ is not limited to the analysis of *S. pneumoniae-*dependent E-cadherin disruption, but rather that it can be broadly applied to assess the health of AJ and TJ components following infection by different respiratory pathogens.

Because IJOQ operates under the assumption that IJs should resemble a mesh-like structure, we expect that this script will accurately quantify the organization of AJ and TJ proteins that are consistent with this localization pattern. As such, we predict that IJOQ can successfully be applied to measure the organization of many AJ proteins (e.g. nectin, catenins, and afadin) and TJ proteins (e.g. occludins, claudins, and junctional adhesion molecules). By extension, IJOQ analysis should be robust across different cell types (e.g. epithelial and endothelial), different tissues (e.g. lung, gut, corneal), and after infection by different pathogens that disrupt this mesh-like pattern (e.g. *B. pertussis, S. aureus, P. aeruginosa*) (Inoshima et al., 2011; Reboud et al., 2017; Hasan et al., 2018). By the same token, we expect IJOQ to struggle quantifying IJs that violate this assumption. For example, gap junctions, another important IJ, are clusters of membrane channels that exhibit a localization pattern that is distinct from a mesh-like structure and thus, not appropriate for IJOQ analysis. Correspondingly, IJOQ does not attempt to measure IJ organization beyond confirming the presence of this mesh-like structure. As a result, it may not detect changes to the phenotype of individual junctions (e.g. continuous, punctate, perpendicular) or of individual gaps in the junctions (e.g. length). Furthermore, while IJOQ is capable of measuring changes to IJs across discrete timepoints (i.e. **Figure 6**), it was not designed to track very rapid dynamic changes of IJs over time (i.e. changes that might be observed across seconds in a video). Lastly, while IJOQ can accept samples that have been co-stained with multiple fluorescent dyes, it is unable to analyze multiple fluorescent channels at once. Based on these design constraints, we do not envision IJOQ as an appropriate image analysis tool to accurately measure very rapid disruptions to IJ organization or to analyze colocalization patterns of IJ proteins. Fortunately, semi-automated fluorescence microscopy analysis programs such as Junction Mapper and Junction Analyzer Program (JAnaP) provide an excellent solution to execute these more detailed types of analysis (Brezovjakova et al., 2019; Gray et al., 2020). These programs provide users with a high level of accuracy for assessing a range of parameters that include individual junction phenotypes, as well as the length, area, and intensity of junction markers (Brezovjakova et al., 2019; Gray et al., 2020). Thus, these programs may offer additional insights into the nature of IJ localization. However, one important limitation of these programs is that they often have more stringent requirements with regard to image quality. For example, Junction Mapper recommends a signal-to-noise ratio above 22, a standard that would exclude many of the images analyzed in this study, which largely range between 10-12 (**Figure 3H**). In contrast, IJOQ efficiently processed all of our images and is capable of accurately analyzing data with a signal-to-noise ratio as low as 0.5 (**Figures 3J, N**). Other important considerations include the opportunity for user bias and speed of analysis. Semi-automated scripts require moderate levels of human input, which creates substantial opportunity for human bias as well as slower analysis speeds. For example, when we performed TiJOR analysis for this study, it could take up to several minutes per image. Conversely, because IJOQ is a fully automated script, human bias is greatly minimized, and it is capable of analyzing an image in 5-10 seconds. Furthermore, the user does not need to be present for the duration of the analysis, as the script automatically parses through the input folder, searches for images to analyze, and saves the measurements.

The steady increase in the use of fluorescence microscopy to describe biological phenomena has created an urgent demand for quantitative image analysis tools to help researchers rigorously assess their data. Our successful creation and application of a fully automated image analysis script to quantify IJ health has identified discrete levels of IJ disruption across different time points, inocula, cell types, and experimental systems. We envision IJOQ and other quantitative imaging methodologies as important resources that will help researchers uncover novel insights in the field of pathogenic microbiology and beyond.

## Data Availability Statement

The raw data supporting the conclusions of this article will be made available by the authors, without undue reservation.

## Ethics Statement

The animal study was reviewed and approved by The Institutional Animal Care and Use Committee at Tufts University.

## Author Contributions

DM and WA contributed to the conception and design of the study. DM, SX, and WA performed experiments. DM, SX, and JR analyzed the data. DM performed the statistical analysis. DM and WA wrote the first draft of the manuscript. DM, SX, SH and WA contributed to manuscript revision, read, and approved the submitted version. All authors contributed to the article and approved the submitted version.

## Funding

This work was supported by the National Institutes of General Medical Sciences Grant 1SC2GM141988-01 (to WA), by the California State University Program for Education and Research in Biotechnology Graduate Student COVID-19 Research Restart Program (to DM). SX was supported by the National Institute on Aging Grant 1 R21 AG071268 awarded to Joan Mecsas and John Leong.

## Conflict of Interest

The authors declare that the research was conducted in the absence of any commercial or financial relationships that could be construed as a potential conflict of interest.

## Publisher’s Note

All claims expressed in this article are solely those of the authors and do not necessarily represent those of their affiliated organizations, or those of the publisher, the editors and the reviewers. Any product that may be evaluated in this article, or claim that may be made by its manufacturer, is not guaranteed or endorsed by the publisher.

## Supplementary Material

Supplementary Figure 1
*B. pertussis* infection of the bronchial epithelium. Shown are the images of **(A)** untreated, **(B)** WT, and **(C)** Δ*cyaA* infections from (Hasan et al., 2018) that were used for IJOQ and TiJOR analysis. The original data and analysis can be found in from the following publication: (Hasan et al. 2018). Bordetella pertussis Adenylate Cyclase Toxin Disrupts Functional Integrity of Bronchial Epithelial Layers. *Infect. Immun.* 86.

## References

[B1] Adams W. Bhowmick R. Bou Ghanem E. N. Wade K. Shchepetov M. Weiser J. N.. (2020). Pneumolysin Induces 12-Lipoxygenase–Dependent Neutrophil Migration During Streptococcus Pneumoniae Infection. J. Immunol. 204, 101–111. doi: 10.4049/jimmunol.1800748 31776202 PMC7195902

[B2] Amieva M. R. Vogelmann R. Covacci A. Tompkins L. S. Nelson W. J. Falkow S. (2003). Disruption of the Epithelial Apical-Junctional Complex by Helicobacter Pylori CagA. Science. (80-.). 300, 1430–1434. doi: 10.1126/science.1081919.Disruption PMC3369828 12775840

[B3] Baschong W. Suetterlin R. Hubert Laeng R. (2001). Control of Autofluorescence of Archival Formaldehyde-Fixed, Paraffin-Embedded Tissue in Confocal Laser Scanning Microscopy (CLSM). J. Histochem. Cytochem. 49, 1565–1571. doi: 10.1177/002215540104901210 11724904

[B4] Bhowmick R. Tin Maung N. H. Hurley B. P. Ghanem E. B. Gronert K. McCormick B. A.. (2013). Systemic Disease During Streptococcus Pneumoniae Acute Lung Infection Requires 12-Lipoxygenase–Dependent Inflammation. J. Immunol. 191, 5115–5123. doi: 10.4049/jimmunol.1300522 24089193 PMC3836588

[B5] Brezovjakova H. Tomlinson C. Mohd-Naim N. Swiatlowska P. Erasmus J. E. Huveneers S.. (2019). Junction Mapper Is a Novel Computer Vision Tool to Decipher Cell-Cell Contact Phenotypes. Elife 8, e45413. doi: 10.7554/eLife.45413 31793877 PMC7034980

[B6] Brückner B. R. Janshoff A. (2018). Importance of Integrity of Cell-Cell Junctions for the Mechanics of Confluent MDCK II Cells. Sci. Rep. 8, 1–11. doi: 10.1038/s41598-018-32421-2 30237412 PMC6148251

[B7] Campbell H. K. Maiers J. L. and DeMali K. A (2017). Interplay Between Tight Junctions & Adherens Junctions. Exp. Cell Res. 358, 39–44. doi: 10.1016/j.yexcr.2017.03.061 28372972 PMC5544570

[B8] Ganesan S. Comstock A. T. and Sajjan U. S (2013). Barrier function of airway tract epithelium. Tissue Barriers 1, e24997. doi: 10.4161/tisb.24997 24665407 PMC3783221

[B9] Garcia M. A. Nelson W. J. Chavez N. (2018). Cell – Cell Junctions Organize Structural and Signaling Networks. Cold Spring Harb. Perspect. Biol 10, 1–28. Availbale at: http://cshperspectives.cshlp.org/content/10/4/a029181. 10.1101/cshperspect.a029181 PMC5773398 28600395

[B10] Gray K. M. Jung J. W. Inglut C. T. Huang H. C. Stroka K. M. (2020). Quantitatively Relating Brain Endothelial Cell-Cell Junction Phenotype to Global and Local Barrier Properties Under Varied Culture Conditions *via* the Junction Analyzer Program. Fluids Barriers CNS 17, 1–20. doi: 10.1186/s12987-020-0177-y 32046757 PMC7014765

[B11] Hasan S. Kulkarni N. Asbjarnarson A. Linhartova I. Osicka R. Sebo P. (2018). Bordetella Pertussis Adenylate Cyclase Toxin Disrupts Functional Integrity of Bronchial Epithelial Layers. Infect. Immun. 86, e00445–17. doi: 10.1128/IAI.00445-17 29203545 PMC5820963

[B12] Heijink I. H. Brandenburg S. M. Noordhoek J. A. Postma D. S. Slebos D. J. van Oosterhout A. J. M. (2010). Characterisation of Cell Adhesion in Airway Epithelial Cell Types Using Electric Cell-Substrate Impedance Sensing. Eur. Respir. J. 35, 894–903. doi: 10.1183/09031936.00065809 19741028

[B13] Inoshima I. Inoshima N. Wilke G. Powers M. Frank K. Wang Y.. (2011). A Staphylococcus Aureus Pore-Forming Toxin Subverts the Activity of ADAM10 to Cause Lethal Infection. Nat. Med. 17, 1310–1314. doi: 10.1038/nm.2451 21926978 PMC3192248

[B14] Jacques L. C. Panagiotou S. Baltazar M. Senghore M. Khandaker S. Xu R.. (2020). Increased Pathogenicity of Pneumococcal Serotype 1 Is Driven by Rapid Autolysis and Release of Pneumolysin. Nat. Commun. 11, 1–13. doi: 10.1038/s41467-020-15751-6 32312961 PMC7170840

[B15] Mathieu C. Mikaty G. Miller F. Lecuyer H. Bernard C. Bourdoulous S.. (2009). Meningococcal Type IV Pili Recruit the Polarity Complex to Cross the Brain Endothelium. Science. (80-.). 325, 83–87. doi: 10.1126/science.1173196 PMC3980637 19520910

[B16] McNeil E. Capaldo C. T. Macara I. G. (2006). Zonula Occludens-1 Function in the Assembly of Tight Junctions in Madin-Darby Canine Kidney Epithelial Cells. Mol. Biol. Cell 17, 1922–1932. doi: 10.1091/mbc.E05 16436508 PMC1415307

[B17] Peter A. Fatykhova D. Kershaw O. Gruber A. D. Rueckert J. Neudecker J.. (2017). Localization and Pneumococcal Alteration of Junction Proteins in the Human Alveolar–Capillary Compartment. Histochem. Cell Biol. 147, 707–719. doi: 10.1007/s00418-017-1551-y 28247028

[B18] Putt K. K. Pei R. White H. M. Bolling B. W. (2017). Yogurt Inhibits Intestinal Barrier Dysfunction in Caco-2 Cells by Increasing Tight Junctions. Food Funct. 8, 406–414. doi: 10.1039/c6fo01592a 28091645

[B19] Rayner C. F. J. Jackson A. D. Rutman A. Dewar A. Mitchell T. J. Andrew P. W.. (1995). Interaction of Pneumolysin-Sufficient and -Deficient Isogenic Variants of Streptococcus Pneumoniae With Human Respiratory Mucosa. Infect. Immun. 63, 442–447. doi: 10.1128/iai.63.2.442-447.1995 7822008 PMC173015

[B20] Reboud E. Bouillot S. Patot S. Béganton B. Attrée I. Huber P. (2017). Pseudomonas Aeruginosa ExlA and Serratia Marcescens ShlA Trigger Cadherin Cleavage by Promoting Calcium Influx and ADAM10 Activation. PloS Pathog. 13, 1–20. doi: 10.1371/journal.ppat.1006579 PMC5584975 28832671

[B21] Schilpp C. Lochbaum R. Braubach P. Jonigk D. Frick M. Dietl P.. (2021). TGF-β1 Increases Permeability of Ciliated Airway Epithelia *via* Redistribution of Claudin 3 From Tight Junction Into Cell Nuclei. Pflugers Arch. Eur. J. Physiol. 473, 287–311. doi: 10.1007/s00424-020-02501-2 33386991 PMC7835204

[B22] Sun Y. Yu H. Zheng D. Cao Q. Wang Y. Harris D.. (2011). Sudan Black B Reduces Autofluorescence in Murine Renal Tissue. Arch. Pathol. Lab. Med. 135, 1335–1342. doi: 10.5858/arpa.2010-0549-OA 21970489

[B23] Takeichi M. (2014). Dynamic contacts: Rearranging adherens junctions to drive epithelial remodelling. Nat. Rev. Mol. Cell Biol. 15, 397–410. doi: 10.1038/nrm3802 24824068

[B24] Terryn C. Sellami M. Fichel C. Diebold M. D. Gangloff S. Le Naour R.. (2013). Rapid Method of Quantification of Tight-Junction Organization Using Image Analysis. Cytom Part A 83 A, 235–241. doi: 10.1002/cyto.a.22239 23212973

[B25] Troeger C. Blacker B. Khalil I. A. Rao P. C. Cao J. Zimsen S. R. M.. (2018). Estimates of the Global, Regional, and National Morbidity, Mortality, and Aetiologies of Lower Respiratory Infections in 195 Countries 1990–2016: A Systematic Analysis for the Global Burden of Disease Study 2016. Lancet Infect. Dis. 18, 1191–1210. doi: 10.1016/S1473-3099(18)30310-4 30243584 PMC6202443

[B26] Waters J. C. (2009). Accuracy and Precision in Quantitative Fluorescence Microscopy. J. Cell Biol. 185, 1135–1148. doi: 10.1083/jcb.200903097 19564400 PMC2712964

[B27] Zhang Y. Wang Y. Cao W. W. Ma K. T. Ji W. Han Z. W.. (2018). Spectral Characteristics of Autofluorescence in Renal Tissue and Methods for Reducing Fluorescence Background in Confocal Laser Scanning Microscopy. J. Fluoresc. 28, 561–572. doi: 10.1007/s10895-018-2217-4 29560601

[B28] Zihni C. Mills C. Matter K. Balda M. S. (2016). Tight junctions: From simple barriers to multifunctional molecular gates. Nat. Rev. Mol. Cell Biol. 17, 564–580. doi: doi:10.1038/nrm.2016.80 27353478

