# Supplementary figures and images for: Dynamic Python-Based Method Provides Quantitative Analysis of Intercellular Junction Organization During S. pneumoniae Infection of the Respiratory Epithelium
Source: Front Cell Infect Microbiol. 2022 Jun 10;12:865528. doi: 10.3389/fcimb.2022.865528 (PMC9230243; doi:10.3389/fcimb.2022.865528)

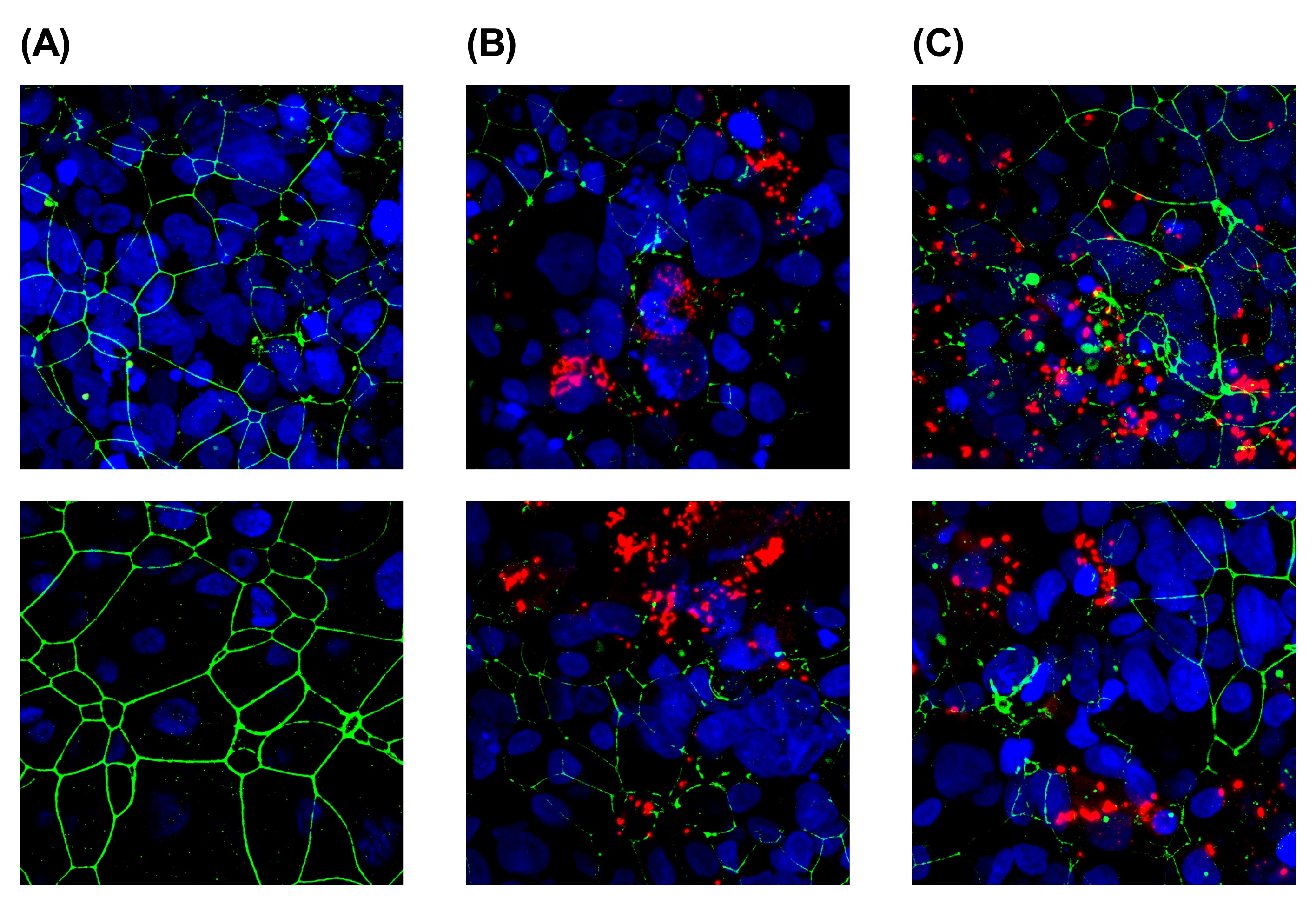

Supplement: Supplementary Figure 1 — B. pertussis infection of the bronchial epithelium. Shown are the images of (A) untreated, (B) WT, and (C) ΔcyaA infections from (Hasan et al., 2018) that were used for IJOQ and TiJOR analysis. The original data and analysis can be found in from the following publication: (Hasan et al. 2018). Bordetella pertussis Adenylate Cyclase Toxin Disrupts Functional Integrity of Bronchial Epithelial Layers. Infect. Immun. 86. [file Image_1.jpg]
